# Purification and biochemical characterization of a novel thermostable protease from the oyster mushroom *Pleurotus sajor-caju* strain CTM10057 with industrial interest

**DOI:** 10.1186/s12896-019-0536-4

**Published:** 2019-07-01

**Authors:** Maroua Omrane Benmrad, Sondes Mechri, Nadia Zaraî Jaouadi, Mouna Ben Elhoul, Hatem Rekik, Sami Sayadi, Samir Bejar, Nabil Kechaou, Bassem Jaouadi

**Affiliations:** 1 Laboratory of Microbial Biotechnology and Engineering Enzymes (LMBEE), Centre of Biotechnology of Sfax (CBS), University of Sfax, Road of Sidi Mansour Km 6, P.O. Box 1177, 3018 Sfax, Tunisia; 2 Biotech ECOZYM Start-up, Business Incubator, Centre of Biotechnology of Sfax (CBS), University of Sfax, Road of Sidi Mansour Km 6, P.O. Box 1177, 3018 Sfax, Tunisia; 3 Laboratory of Environmental Bioprocesses (LEBP), LMI COSYS-Med, Centre of Biotechnology of Sfax (CBS), University of Sfax, Road of Sidi Mansour Km 6, P.O. Box 1177, 3018 Sfax, Tunisia; 4 Research Group of Agro-Food Processing Engineering (GP2A), Laboratory of Applied Fluid Mechanics, Process Engineering and Environment, National School of Engineers of Sfax (ENIS), University of Sfax, Road of Soukra Km 4, P.O. Box 1173, 3038 Sfax, Tunisia

**Keywords:** Protease, Oyster mushroom, *Pleurotus sajor-caju*, Organic solvent, Peptide synthesis, Detergent formulations

## Abstract

**Background:**

Proteases are hydrolytic enzymes that catalyze peptide linkage cleavage reactions at the level of proteins and peptides with different degrees of specificity. This group draws the attention of industry. More than one protease in three is a serine protease. Classically, they are active at neutral to alkaline pH. The serine proteases are researched for industrial uses, especially detergents. They are the most commercially available enzyme group in the world market. Overall, fungi produced extracellular proteases, easily separated from mycelium by filtration.

**Results:**

A new basidiomycete fungus CTM10057, a hyperproducer of a novel protease (10,500 U/mL), was identified as *Pleurotus sajor-caju* (oyster mushroom). The enzyme, called SPPS, was purified to homogeneity by heat-treatment (80 °C for 20 min) followed by ammonium sulfate precipitation (35–55%)-dialysis, then UNO Q-6 FPLC ion-exchange chromatography and finally HPLC-ZORBAX PSM 300 HPSEC gel filtration chromatography, and submitted to biochemical characterization assays. The molecular mass was estimated to be 65 kDa by sodium dodecyl sulfate polyacrylamide gel electrophoresis (SDS-PAGE), Native-PAGE, casein-zymography, and size exclusion by HPLC. A high homology with mushroom proteases was displayed by the first 26 amino-acid residues of the NH_2_-terminal aminoacid sequence. Phenylmethanesulfonyl fluoride (PMSF) and diiodopropyl fluorophosphates (DFP) strongly inhibit SPPS, revealing that it is a member of the serine-proteases family. The pH and temperature optima were 9.5 and 70 °C, respectively. Interestingly, SPPS possesses the most elevated hydrolysis level and catalytic efficiency in comparison with SPTC, Flavourzyme® 500 L, and Thermolysin type X proteases. More remarkably, a high tolerance towards organic solvent tolerance was exhibited by SPPS, together with considerable detergent stability compared to the commercial proteases Thermolysin type X and Flavourzyme® 500 L, respectively.

**Conclusions:**

This proves the excellent proprieties characterizing SPPS, making it a potential candidate for industrial applications especially detergent formulations.

## Background

Enzymes are natural catalysts, which are increasingly more required in various industrial fields and bioprocesses. Today, researchers focus on detecting new enzymes endowed with extremely satisfactory properties for commercial applications [1, 2]. Using enzymes in industrial process has a large number of advantages, namely their specificity and lower pressures, i.e. lower cost. Besides, they can be preserved for a long time. They are also biodegradable, therefore causing less environmental pollution. Global demand for industrial enzymes is expected to rise up to almost 7.1 billion dollars in 2018, www.FreedoniaGroup.com (Cleveland, USA). Compared to plant and animal enzymes, microbial enzymes are the most attractive ones to industrialists owing to their ease of production [3]. In fact, the microbial proteases production is beneficial thanks to their distinctive features such as short generation time, easy manipulation of microorganisms genes, and large availability in nature [1]. Besides, their preferred properties in biotechnology are widely approved. Proteases isolated from plant and animal do not receive industrial demand save for some uses. Around three quarts of industrial biocatalysts are hydrolyses [4, 5], 65% of them are proteases of the total industrial enzyme market [6, 7]. The business applications of proteases are quickly growing thanks to the current technological advances. These hydrolyzes possess have original properties and substrate specificities [8]. They have been well-studied, being considered as the most attractive group [9]. The majority of the total sales (89%) of protease are used in detergent formulation [10]. Historically, proteases were the first to be used in laundry detergents [2, 11, 12], about two-third share of this industry [13, 14]. Thanks to these enzymes, detergent industries have made considerable progress [11, 13]. It is noteworthy that protease is a hydrolytic biocatalyst which catalyzes the interruption of protein polymers [9, 12, 15, 16]. It contributes significantly in the metabolism of almost all organisms [4]. Besides, bio-additives improving the efficiency and the performance of laundry detergents are receiving increasing demands. In fact, a large number of researches are focusing on the isolation and purification of alkaline proteases for detergence purpose. Although industrial applications resort to bacterial proteases, the process of cell elimination is expensive, thus their use is restricted [17]. It is noteworthy that bacteria produce the majority of proteases of microbial origin. In fact, *Bacillus* genus produces neutral and alkaline proteases. The excellent properties of basidiomycetes’ proteases pave the way for further study. Although the 3D structural and functional proprieties of basidiomycetes proteases occurred more than 30 years ago in scientific research, the variety and complexity of xylotrophic basidiomycetes enzymes action has resulted in interesting studies. In this respect, our researches are focused on fungi protease thanks to their easy cell separation. The study of micromycetes proteases has recently shed lights on the specific characteristics of enzymes [5, 18]. Moreover, basidiomycota are a secret treasure proteolytic variety [19]. Several species of strains including fungi (viz. *Aspergillus flavus*, *Aspergillus clavatus*, *Aspergillus melleu*, *Aspergillus niger*, *Fusarium graminarum*, *Chrysosporium keratinophilum*, *Penicillium griseofulvin*, *Penicillium chrysogenum, Scedosporium apiosermum*, and *Trametes cingulata*) and bacteria (viz. *Bacillus licheniformis*, *Bacillus amyloliquefaciens*, *Bacillus pumilus*, *Bacillus safensis*, *Bacillus firmus*, *Bacillus alcalophilus*, *Bacillus proteolyticus*, *Bacillus subtilis*, and *Bacillus thuringiensis*) are described as foodstuffs proteases [6, 12, 20, 21]. However, the fungi protease origin, *Penicillium* and *Aspergillus* are widely studied, as a greater species and can produce different proteases types, such as *Aspergillus oryzae*. In fact, mushroom species have been proven to possess various biological effects. For centuries, mushrooms have been used as food and food materials, as well as being topics of study in many fields, namely medicine and cosmetics. Oyster mushrooms, particularly, represent a type of specialty mushroom that is much prized around the world and most popularly in the United States. Indeed, mushrooms are a source of protein, vitamins C and B, and minerals, and mainly endowed with a very low fat content [22]. Many bioactive molecules, including protease, have been isolated from superior fungi. More than twenty proteases have been isolated from fungi species [23]. Moreover, the study of basidiomycota’s proteases is still limited. Thus, the study of new *Pleurotus* proteases is a biotechnology area that necessitates to be discovered. The *Pleurotus* species are extremely valued in cooking thanks to their refined flavor and they have also been examined in different bioactive molecules such as antitumor, hypocholesterolemic, anti-inflammatory antiviral, antioxidant, antibiotic, antidiabetic, antitumor, immunomodulatory, antihyperlipidemic, and hepatoprotective compounds etc. [1]. Numerous researches have demonstrated the fibrinolytic activity of edible mushrooms, including *Flammulina velutipes*, *Pleurotus ostreatus*, *Grifola frondosa*, *Tricholoma saponaceum*, and *Armillaria mellea* [23]. The genus *Pleurotus* is known for laccase production. This is linked to the ligninolytic metabolism with the presence of proteases. In addition to laccase production, this genus is known for the hydrolytic enzymes production by *Pleurotus* spp. that have been designated, resulting in these properties of this genus for future research [1].

The study of saprophytic basidiomycetes proteases has led to the isolation of different classes of proteases [19]. As white rot fungi, the genus *Pleurotus* has ligninolytic [24] and proteolytic [1, 5, 19] enzymes. A few studies have investigated the proteolytic enzymes isolated from *Pleurotus* spp. as reported. This microorganism is a white rot fungi and the second crucial group of fungi cultivated in life, with more than 30 species [1]. It is the most produced edible mushroom, withstanding any temperature. It was found in tropical and subtropical regions and can grow artificially with no difficulties. This genus can be cultivated at any substrate and temperature. In fact, its easy cultivation is performed by means of cheap nutriments. Besides, it is characterized by excellent adaptation [5, 25], and therefore, a high yield of production.

In this study, a novel alkaline and detergent-stable protease, called SPPS, from the fungus *Pleurotus sajor-caju* strain CTM10057, was purified to homogeneity and its enzymatic characteristics were examined. Moreover, the enzymatic performance evaluation of the SPPS was compared with other used proteases. This present work provides valuable information on biochemical characteristics of SPPS from the oyster mushroom.

## Methods

### Substrates, chemicals, and used comparative proteases

All the chemicals and substrates used in this study were reagent position, unless specified otherwise. General reagents were obtained from commercial suppliers. The SPTC enzyme is a fungi protease from *Trametes cingulata* CTM10101 [26]. The Flavourzyme® 500 L is a commercial fungal proteolytic enzyme from *Aspergillus oryzae* supplied by Novozymes A/S (Bagsvaerd. Denmark). The Thermolysin type X is a commercial protease from *Geobacillus stearothermophilus* purchased from Sigma Aldrich Inc., Fluka, Chemical Co. (St. Louis, MO, USA).

### Isolation and growth culture conditions of protease-producing white-rot-fungi and enzyme production

Fruiting bodies of a wild *Pleurotus* and *Shiitake* were collected from different biotope sites, from the symptomatic wood of the camphor trees *Quercus faginea* (L.) at the El Feïdja National Park (Aïn Draham, Jendouba, Tunisia) to isolate protease-producing fungi. Mycelium pure cultures of the *Pleurotus* P10 (designated as CTM10057) were obtained in Petri dishes, containing potato dextrose agar (PDA) medium at 28 ± 2 °C and pH 6.0. The plates were then incubated for 7 d at 28 ± 2 °C. To avoid bacterial growth, the plates were supplemented with chloramphenicol 25 μg/mL. The protease-producing fungal strain was tested onto skimmed milk agar plates containing (g/L): peptone, 5; yeast extract, 3; skimmed milk 250 mL; and bacteriological agar, 20 at pH 7.4. The protease-producing strain was propagated on PDA plates at 28 ± 2 °C, and the inoculates were prepared from 6-day-old colonies by flooding with 10 mL of sterile distilled water and scraping off the agar plates. 2 mL of homogenized mycelium were used for inoculation of 1000-mL Erlenmeyer flask with baffles, containing 100 mL of culture medium. This basal medium incorporates a complete potato dextrose broth (PDB) liquid culture medium supplemented with (g/L): 15 lentil flour; 3 yeast extract; 10 glucose; 1 KH_2_PO_4_; 1 K_2_HPO_4_ at pH 5.6. Cultures were incubated for 3 d at 28 °C on a rotary shaker with a shaking speed of 180 rpm. Afterwards, the culture broths were centrifuged at 11000×*g* for 30 min to remove mycelia and medium debris. The cell-free supernatant was designated as crude enzyme.

### PCR amplification and ITS of 18S rDNA gene sequencing and molecular phylogenetic investigation

The genomic DNA of the isolate fungal strain CTM10057 was extracted from freeze-dried mycelia according to CTAB method as investigated previously by Li [27]. The gene encoding ITS region of rDNA was amplified by the fungi identification PCR kit by means of universal primers ITS1 and ITS4 as described elsewhere [13]. The PCR product was placed into the PCR2.1 vector by means of a pCR-Blunt ended cloning kit (GE Healthcare/Amersham Biosciences Corp., Piscataway, NJ, USA). The nucleotide sequences of both strands pertaining to the cloned ITS rDNA gene sequence were sequenced in both directions via universal primers (U-19 and T7) using the automated DNA sequence ABI PRISM® 3100-Avant Genetic Analyzer (Applied Biosystems, Foster City, CA, USA). The nucleotide sequence alignment was conducted several times, using the BioEdit (v 7.0.2) software. The analyses of phylogenetic and molecular evolutionary were carried out using Molecular Evolutionary Genetics Analysis (v 4.1) program.

### Determination of protease activity

The SPPS activity was measured spectrophotometrically at 660 nm using casein as substrate [28] at 70 °C and 100 mM glycine-NaOH buffer added with 2 mM CaCl_2_ at pH 9.5 (i.e., Buffer A), under the standard assay conditions described previously [26].

The SPPS activity, in detergent solution, was measured at 450 nm using *N*,*N*-dimethylated casein as substrate at 40 °C and pH 9 as reported elsewhere [29].

### SPPS purification procedure

To remove microbial cells, five hundred mL of a 72-h culture of *Pleurotus sajor-caju* strain CTM10057 were centrifuged. The supernatant containing extracellular protease was used as the crude enzyme preparation and submitted to the following purification steps. First, the filtrate was incubated for 20 min at 80 °C and insoluble material was removed by centrifugation at 11000×*g* for 20 min. The ammonium sulfate was added to clear supernatant to reach 35% saturation, then centrifuged at 11000×*g* for 30 min. The saturation of the obtained supernatant was done up to 55% with solid (NH4)_2_SO_4_, re-centrifuged, re-suspended in a minimal volume of 50 mM PIPES at pH 6.1 (i.e., Buffer B), and dialyzed overnight against repeated changes of the same buffer. The sample, thus, obtained was deposited on a UNO Q-6 column (12 mm × 53 mm), using FPLC system, previously equilibrated with buffer B. The proteins were eluted with the same buffer, containing an increasing concentration of NaCl of 0 to 500 mM at a rate of 0.5 mL/min. Fractions of each peak were collected manually and analyzed by measuring absorbance at 280 nm and the proteolytic activity on casein. Pooled fractions, containing protease activity, were further resolved by HPLC system using ZORBAX PSM 300 HPSEC (6.2 mm × 250 mm), Agilent Laboratories, pre-equilibrated with 25 mM HEPES buffer at pH 7.5, were supplemented with 2 mM CaCl_2_ (i.e., Buffer C). Protein was separated by molecular weight at a flow rate of 0.5 mL/min with buffer C and the protease activity was detected using a UV spectrophotometric detector at 280 nm.

### Proteins measurement, electrophoresis, and analytical methods

Protein concentration was measured as reported by Bradford [30], using BSA as reference. Previously reported by Laemmli [31], the subunit molecular weight of the purified SPPS enzyme was evaluated by SDS-PAGE under reducing conditions, using 12% separating gel (pH 8.8) and 5% stacking gel (pH 6.8). The Native-PAGE or non-reducing conditions of the purified SPPS was performed using 12% resolving gel (stacking gel was omitted) in Tris-glycine buffer (pH 8.5) at 4 °C. The molecular weight of the purified SPPS protein was determined in comparison with standard protein markers and also confirmed by size exclusion HPLC, using ZORBAX PSM 300 HPSEC (Agilent Technologies, Lawrence, Kansas, MO, USA), pre-equilibrated with the buffer C. The protein bands were visualized with Coomassie Brilliant Blue R-250 (Bio-Rad Laboratories, Inc., Hercules, CA, USA) staining. Substrate-PAGE, or casein zymography staining, was performed by incorporating azo-casein into the separating gel before polymerization as detailed by Jaouadi et al. [32]. The gels were rinsed twice for 30 min in Triton X-100 (2.5%), after electrophoresis, in order to remove the SDS. The occurrence of clearance zone around the protein band on the blue background elucidated the SPPS protease activity.

### Edman degradation for amino-acid sequencing

To sequence NH_2_-terminal, the bands of purified SPPS enzyme were cut from SDS gels and transferred to a ProBlott membrane (Applied Biosystems, Foster City, CA, USA). Accordingly, in order to perform such analysis, we used automated Edman’s degradation at the Faculty of Science of Sfax (FSS), University of Sfax, by means of a protein sequencer (Applied Biosystems Protein sequencer ABI Procise 492/610A).

### Biochemical characterization of the purified SPPS enzyme

#### Effects of inhibitors and metallic ions on enzyme stability

The effects of some inhibitors and various divalent metallic ions on protease stability were examined by means of pre-incubating the purified SPPS enzyme for 1 h at 40 °C, in the presence of each inhibitor or metallic ions, in order to characterize the SPPS-owned class. Performing enzyme assays required standard assay conditions.

#### Influence of pH on the activity and stability of SPPS

The influence of pH was carried out with casein at 10 g/L as a substrate. Protease activities were studied over a pH range [2.0–13.0] at 70 °C. For the measurement of pH stability, the purified SPPS enzyme was pre-incubated in buffers at different pH in the range of 2.0–13.0 for 24 h at 40 °C. Aliquots were withdrawn periodically and the residual proteolytic activities were measured as aforementioned.

#### Effects of temperature on the enzyme activity and stability

With respect to proteolysis activity, its optimum temperature was determined via performing SPPS at pH 9.5 at different temperatures from 40 to 100 °C, with and without 2 mM CaCl_2_. At a pH of 9.5, the thermostability was determined by incubating SPPS at 80, 90, and 100 °C for 12 h of incubation with and without 2 mM CaCl_2_. Aliquots were withdrawn at desired time intervals to test the remaining activity under standard conditions. Considered as control (100%), the non-heated SPPS was left at room temperature. Half-life times of the purified SPPS protease were determined after incubation at 80, 90, and 100 °C for 12 h.

#### Effect of various polyol(s) and /or calcium on SPPS thermostability

The effect of variety of polyols, with concentration of 100 mg/mL, on SPPS thermostability was carried out by its incubation at 90 °C for 1 h with and without different polyols. The combination of sorbitol (100 mg/mL) and calcium (2 mM) on the thermostability of SPPS enzyme was monitored at 90 °C for 12 h. Protease assays were carried out under the standard experimental assay conditions.

### Kinetic study of the SPPS, SPTC, Flavourzyme® 500 L, and Thermolysin type X enzymes

#### Substrate specificities profile

The substrate profile specificity of SPPS was determined using natural and modified protein substrates as well as esters substrates. Enzymatic activities were determined on each substrate based on standard conditions, previously described elsewhere [33, 34].

#### Kinetic measurements

The kinetic constants for protease activity were determined by performing casein as substrate at different concentrations, ranging from 0.10 to 50 mg/mL for 20 min. The pH and temperature values used in the kinetic study were adjusted to the optimum conditions for each enzyme (SPPS, pH 9.5 and 70 °C; SPTC, pH 9.0 and 60 °C; Flavourzyme® 500 L, pH 11.0 and 60 °C; and Thermolysin type X, pH 8.0 and 70 °C). The purified enzymes used were SPPS; SPTC; Flavourzyme® 500 L, and Thermolysin, at a final concentration of proteins 1.5 mg/mL using natural (casein) as specific substrates for all proteases. Each assay was carried out in triplicate, and Lineweaver–Burk plots were used in order to rate kinetic parameters. The Hyper32 v.1.0. software was used to calculate Kinetic constants, Michaelis–Menten constant (*K*_m_), and maximal reaction velocity (*V*_max_) values.

### Performance evaluation of the purified SPPS, SPTC, Flavourzyme® 500 L, and Thermolysin type X enzymes

#### Determination of hydrolysis degree with casein

Casein hydrolysis was carried out at 70 °C and pH 9.5 for SPPS; at 60 °C and pH 9.0 for SPTC; at 70 °C and pH 8.0 for Thermolysin type X; and at 60 °C and pH 11.0 for Flavourzyme® 500 L. So, an amount of 5 g of casein was dissolved in 100 mL of 100 mM buffer per used enzyme and then treated with 500 U of the purified enzymes (SPPS, SPTC, Flavourzyme® 500 L, and Thermolysin type X). For each case, the degree of hydrolysis (DH) was calculated based on the amount of base (NaOH) added so that the pH remains constant during hydrolysis [35] as previously reported by Zaraî Jaouadi [34]. The DH is defined as the percentage of peptide bonds cleaved according to the total number of peptide bonds, i.e. it is the ratio of free amino-acids to those in peptide linkage and the relative size of the peptide fragments [36]. By totally decomposing the protein into its constituent elements, this degree of hydrolysis is estimated to be 100%. Since enzymes are specific biocatalysts, no enzyme alone is capable of hydrolyzing each peptide bond of a protein up to 100% DH. Indeed, the DH of an enzyme provides us with an idea of its functionality. Hydrolysis helped to keep the pH titrimetrically constant by using the pH-Stat (Metrohm 718 Stat Titrino, Herisau, Switzerland) set at the desired pH value of each enzyme via constantly adding 5 N of NaOH (caustic soda) [35, 37].

#### Effects of organic-solvents on protease stability

Various organic solvents, with different log P_ow_ values at 50% (v/v), were tested by shaking at 150 strokes per min and 37 °C for 72 h to evaluate their effects on SPPS and Thermolysin type X protease stabilities. The polarity or hydrophobicity of an organic solvent was quantified by a parameter termed “log P_ow_” value. Generally, organic solvents, using low log P_ow_ values, show more biotoxicity and result in the inhibition of the biocatalyst compared with high log P_ow_ solvents [38]. The relative and residual caseinolytic activities were assayed under each assay condition at 70 °C and pH 9.5 (for SPPS) and at 60 °C and pH 8.0 (for Thermolysin type X). Devoid of any organic solvent, the activity of SPPS was taken as 100%.

#### Compatibility of SPPS enzyme with some commercial detergents

The stability of the alkaline proteases (SPPS, Flavourzyme® 500 L, and SPTC) in the presence of miscellaneous laundry detergents was examined by incubating enzymes (500 U/mL) for 1 h at 40 °C with various common detergent preparations (7 mg/mL), and the residual activities were determined. Prior to the addition of each purified enzyme, the endogenous enzymes contained in used detergents were deactivated by incubating the diluted detergents at 65 °C for 1 h. Under similar incubation conditions, the enzyme activity of a control (devoid of any detergent) was taken as 100%.

#### Washing performance evaluation of SPPS enzyme

The application of the proteases SPPS and Flavourzyme® 500 L at 500 U/mL as a detergent bio-additive in Dixan detergent (7 mg/mL) were studied on white cotton cloth pieces stained with blood and evaluated as already detailed by the authors [26].

#### Culture collection depository number and nucleotide sequence accession number

The fungal culture of P10 isolate was deposited in the « Collection Tunisienne de Microorganismes (CTM) » at the Service of Culture Collection Maintenance of the Centre of Biotechnology of Sfax (Sfax, Tunisia) under the authentic culture collection number: CTM < TUN>:10057 (designated as CTM10057). The data reported in this work for the nucleotide sequences of the 18S rDNA (750 bp) gene were deposited in the GenBank/ENA/EMBL databases under accession number: MH806376.

## Results

### Screening of alkaline protease-producing strains

**Fig. 1:**
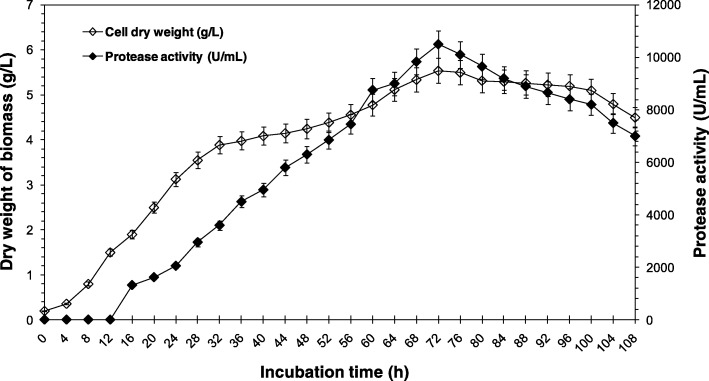
Time course of *Pleurotus sajor-caju* strain CTM10057 biomass (dry weight of mycelia) (◊) and protease activity (production) (♦) on optimized culture medium. Cell growth was performed by measuring the dry matter. Protease activity was determined in culture filtrates obtained after removal of cells by centrifugation, as described in Methods section. Each point represents the mean (*n* = 3) ± standard deviation

The new basidiomycete fungus, *Pleurotus* P10, was newly isolated from the symptomatic wood of the camphor trees *Quercus faginea* (L.) at the El Feïdja National Park, Aïn Draham (Jendouba, Tunisia). It was registered in the CTM-CBS under the authentic culture number: “CTM10057” and was retained, for further and advanced study, as the highest protease producer fungal strain on skimmed milk agar after 24 h (with diameter of clear zone/ diameter of colony growth > 3 mm) and on PDB liquid medium after 72 h (Fig. 1) (with protease activity = 10,500 U/mL) at 28 ± 2 °C.

### Identification and molecular phylogeny of the microorganism

In order to identify CTM10057 strain, the internal transcribed spacer region of 18S rDNA (750 bp) was amplified, cloned in PCR2.1 vector and sequenced. The ITS nucleotide sequence was analyzed with the GenBank database using BLAST program and showed 99 and 98% homology with ITS of *Pleurotus sajor-caju* MUCL:40167 (accession no.: JN645075) and *Trametes marianna* BJFC12714 (accession no.: KC848334) strains, respectively. The phylogenetic tree was established by the neighbor-joining method (data not shown). The multiple alignment and phylogenetic tree revealed that CTM10057 strain is *Pleurotus sajor-caju* (accession no.: MH806376).

### Purification of SPPS enzyme

**Fig. 2:**
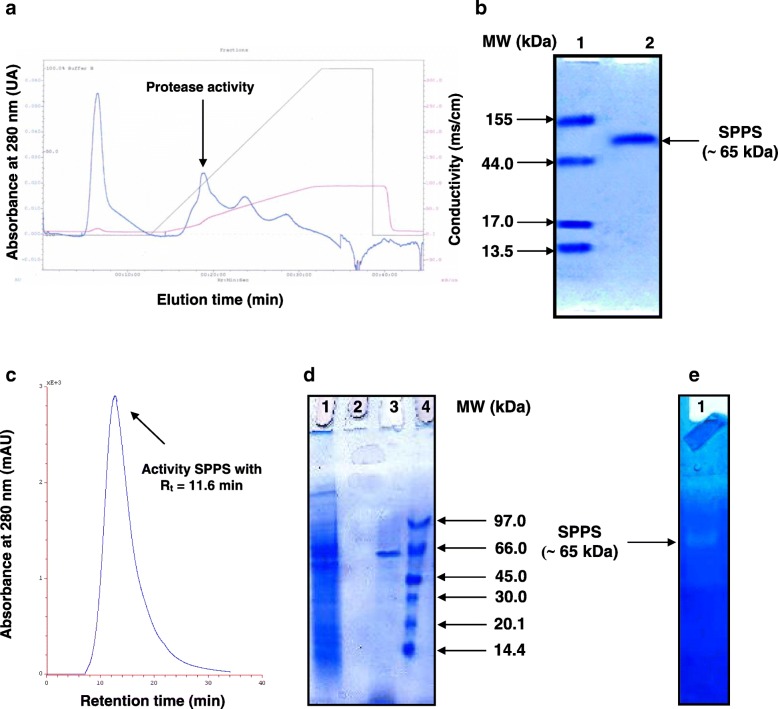
Purification and identification of the protease SPPS from *Pleurotus sajor-caju* strain CTM 10057. **a** Chromatography profile of the partial protease purification on FPLC system using UNO Q-6. The column (12 mm × 53 mm) (Bio-Rad Laboratories, USA) was equilibrated with buffer C. The adsorbed material was eluted with a linear NaCl gradient (0 to 500 mM in buffer C at a flow rate of 30 mL/h, as described in Methods section. **b** The assessment of homogeneity and molecular weight analysis of purified SPPS protein on Native-PAGE, Lane 1, protein markers (molecular masses in kDa). Lane 2, purified SPPS enzyme from *Pleurotus sajor-caju* strain CTM 10057 (30 μg). **c** Chromatography profile of the purified protease SPPS on HPLC system using ZORBAX PSM 300 HPSEC. The column (6.2 mm × 250 mm) (Agilent Technologies, Lawrence, Kansas, MO, USA) was equilibrated with buffer C and native protein markers of 670, 158, 44, 17, and 13.5 kDa, show a single peak of 65 kDa, approximately. Proteins were separated by isocratic elution at a flow rate of 30 mL/h with buffer C and detected using a UV-VIS Spectrophotometric detector (Knauer, Berlin, Germany) at 280 nm. The pure SPPS enzyme, with retention time (R_t_) of 11.6 min, contains protease activity. **d** SDS-PAGE 12% of the purified protease. Lane 1, Total cell extract. Lane 2, Empty. Lane 3, Purified SPPS (30 μg) obtained after HPLC-ZORBAX PSM 300 HPSEC chromatography at R_t_ = 11.6 min. Lane 4: Amersham LMW protein marker (GE Healthcare Europe GmbH, Freiburg, Germany). **e** Zymogram caseinolytic activity staining of the purified protease SPPS (30 μg)

The SPPS enzyme was purified from the culture supernatant according to the procedure described in the Methods section. The supernatant was obtained by the centrifugation of a 72-h old culture of the *Pleurotus sajor-caju* strain CTM10057 (Fig. 1) using broth (500 mL) as a crude enzyme solution. The protein elution profile obtained at the final purification step indicated that the protease was eluted at 110–160 mM NaCl (Fig. 2a). Fractions corresponding protease activity were loaded in HPLC column.

The results of the purification procedure are summarized in Table 1. SPPS purified enzyme preparation contained about 15% of the total activity of the crude and had a specific activity of 79,000 U/mg (Table 1).

**Table 1:** Flow sheet purification of SPPS from *Pleurotus sajor-caju* strain CTM10057

Purification step ^a^	Total activity (units) ^b^ × 10^4^	Total protein (mg) ^b.c^	Specific activity (U/mg of protein) ^b^	Activity recovery rate (%)	Purification factor (fold)
Crude extract	525 ± 13	590 ± 14	8823	100	1
Heat treatment (20 min at 80 °C)	430 ± 10	240 ± 6	17,917	82	2
(NH_4_)_2_SO_4_ fractionation (35–55%)-dialysis	375 ± 9	160 ± 4	23,437	71.4	3
FPLC (UNO Q-6)	152 ± 3	40 ± 1	38,000	28.5	4.3
HPLC (ZORBAX PSM 300 HPSEC)	79 ± 2	10 ± 0.5	79,000	15	9

^a^ Experiments were conducted three times and ± standard errors are reported

^b^ One unit (U) of protease activity was defined as the amount of enzyme required to release 1 μg tyrosine per minute under the experimental conditions used

^c^ Amounts of protein were estimated by the method of Bradford [30]

### Molecular weight determination of SPPS enzyme

To analyze the homogeneity and molecular weight of the purified SPPS enzyme, Native-PAGE and SDS-PAGE were performed. As shown in Fig. 2b, the molecular mass of the SPPS was almost 65 kDa, as assessed by Native-PAGE. Moreover, the SPPS preparation was a homogeneous pure enzyme with high purity confirmed by a symmetrical peak with retention time (R_t_) of 11.6 min, matching with protein of ~ 65 kDa (Fig. 2c) reached after native condition in HPLC chromatography (Fig. 2c). Under reducing conditions (SDS-PAGE), a unique monomeric protein band estimated to 65 kDa was obtained for the purified SPPS enzyme (Fig. 2d) corresponding to that determined by gel filtration. Purity of the SPPS protease was also evaluated by zymography. The zymogram activity staining confirms the presence of a clear zone of protease activity for the purified sample and total cell extract co-migrating with proteins, having molecular masses of 65 kDa (Fig. 2e).

These observations strongly suggested that SPPS was a monomeric protein comparable to those previously reported for other fungi proteases [25, 26, 33, 39].

### NH_2_-terminal amino-acid sequence determination of SPPS enzyme

The sequence of the first 26 NH_2_-terminal amino-acid residues of SPPS from the *Pleurotus sajor-caju* strain CTM10057 was determined as: GPEDPALPPDSESTHVITGVEKLHAQ. It showed uniformity, thus indicating that it was isolated in a pure form. This sequence was submitted to comparisons with existing protein sequences in the GenBank non-redundant protein database and the Swiss-Prot database, using the BLASTP and tBlastn search programs (Table 2). The alignment of the NH_2_-terminal amino-acid shows that this sequence has homology with the subtilisin-like proteases; or this group of subtilisin-like proteases is found in bacteria, mammals, insects, and in some yeasts. This protease class exhibits significant homology with the protease of basidomycetes fungi, which consist of 300 to 400 amino-acid residues and do not have disulfide bridges as well as being characterized by the absence of cysteine and cystine residues. They are involved in various functions depending on the species, having in most cases nutritive functions. The effect of calcium as a stabilizer was demonstrated in some cases. It appears that the Ca^2+^ ion binds and gives the enzyme a more or less rigid structure, thus decreasing auto-digestion. The NH_2_-terminal sequence displayed a medium to significant similarity (64–90%) compared to that of fungi serine proteases. Table 2 represents the NH_2_-terminal sequence of SPPS, which was compared to other closely related proteolytic sequences, noted to share greatest homology with fungal proteolytic enzymes, especially those from *Pleurotus ostreatus* strain ATCC® MYA-2306™ (90% similarity with SPPS). The latter contains one modified amino-acid. The Glu3 residue in SPPS was substituted by Asp3. Besides, SPPS showed 85, 79, 75, and 64% similarities with proteases from *Schizopora paradoxa* strain KUC8140, *Phlebia centrifuga* strain FBCC195, *Phanerochaete chrysosporium* strain S1, and *Metarhizium anisopliae* strain ARSEF 549, respectively. The NH_2_-terminal amino-acid of SPPS sequence differed from protease PR1C from *Metarhizium anisopliae* strain ARSEF 549 (64% similarity with SPPS) by fifteen amino-acid residues at positions G1L, P2S, E3S, D4R, A7D,  L8D, P8G, P9A, S11I, E12F, T14P, I17M, E21K, and K22L. These results and the comparison with the closest enzymes indicated that SPPS proteolytic enzyme is a new member of the serine protease from fungal strains family.

**Table 2:** Alignment of the NH_2_-terminal amino-acid sequence of the purified SPPS from *Pleurotus sajor-caju* strain CTM10057 with the sequences of other fungal proteases

Enzyme	Origin	NH_2_-terminal amino-acid ^a,b^	Identity (%)
SPPS (this work)	*Pleurotus sajor-caju* strain CTM10057	GPEDPALPPDSESTHVITGVEKLHAQ	–
PoSl	*Pleurotus ostreatus* strain ATCC® MYA-2306™	GP **D** DPALPPD	90
PA_PoS1	*Schizopora paradoxa* strain KUC8140	GP **T** DPA **I** PPDSESTHV **L** TGV	85
VPR	*Phlebia centrifuga* strain FBCC195	**DDPAI** PPD **T** ESTHV **L** TGV **D** KLHAQ	79
PcSl	*Phanerochaete chrysosporium* strain S1	G **KS** DPA **V** P **A** D **T** ESTH	75
PR1C	*Metarhizium anisopliae* strain ARSEF 549	**LSSR** A **DDGA** D **IF** S **P** HV **MTQV** KL	64

^a^ Amino-acid sequences for comparison were obtained using the program BLASTP (NCBI, NIH, USA) database with default parameters

^b^ Residues not identical with SPPS protease from *Pleurotus sajor-caju* strain CTM10057 are indicated in black box

### Biochemical proprieties of SPPS enzyme

#### Influence of enzyme inhibitor and metal ions

In order to determine the nature of SPPS enzyme, the effect of several enzyme inhibitors, (chelating agent and a specific group reagent) [7] on enzyme stability was investigated (Table 3). Trypsin-like and chymotrypsin competitive reagents did not have any influence on the stability of the purified SPPS. However, SPPS was thoroughly inhibited by the serine protease inhibitors, indicating that this enzyme belongs to that subclass. Metallo-enzyme inhibitors EDTA and EGTA sparsely affect the SPPS stability. EDTA at 1 mM and EGTA at 2 mM inhibited SPPS only by 91 and 80% of its activity, respectively. The relative stability of this enzyme in the presence of EDTA and EGTA is beneficial and auxiliary for use as detergent bio-additive, since detergents formulation contain high portion of chelating agents, which function as water softeners and further facilitate the proteinacious stain removal. Other metallic ions were also assayed for their effects on SPPS activity (Table 3). This fungi protease enzyme is totally activated by Ca^2+^, Mg^2+^, and Fe^2+^ but strongly inhibited by Cd^2+^, Ni^2+^, and Hg^2+^ (2 mM).

**Table 3:** Effects of various inhibitors, reducing agents, and metallic ions on SPPS enzyme stability. Protease activity measured in the absence of any inhibitor or reducing agent was taken as control (100%). The non-treated and dialyzed enzyme was considered as 100% for metallic ion assay. Residual activity was measured at pH 9.5 at 70 °C

Reagent		Specificity/origin	Concentration	Relative protease stability (%) ^a^
*Inhibitor/reducing agents*	Control		–	100 ± 2.5
	PMSF or DFP	Serine protease specific inhibitors	5 mM	0 ± 0.0
	Benzamidine	Competitive inhibitor of serine proteases	10 mM	103 ± 2.6
	Aprotinin		5 mM	101 ± 2.5
	SBTI	Soybean trypsin inhibitor	3 mg/mL	101 ± 2.5
	TPCK	Chymotrypsin specific inhibitor	1 mM	94 ± 2.2
	TLCK	Trypsin-like specific inhibitor	1 mM	102 ± 2.5
	DTNB	Cysteine or thiol-containing protease inhibitors	5 mM	41 ± 0.8
	NEM		5 mM	74 ± 1.8
	MIA		10 mM	97 ± 2.4
	EPNP		2 mM	87 ± 2.1
	Iodoacetamide		50 μg/mL	65 ± 1.4
	β-ME	Thiol reducing agent for cleaving protein disulfide bonds (cystin)	1 mM	66 ± 1.6
	ld-DTT		50 μM	61 ± 1.5
	EDTA	Specific metallo-protease inhibitors	1 mM	91 ± 2.2
	EGTA		2 mM	80 ± 2.1
	1,10-Phenanthroline monohydrate		10 mM	102 ± 2.5
	Leupeptine	Acid protease specific inhibitors	10 μg/mL	101 ± 2.5
	Pepstatine A		10 mM	104 ± 2.7
*Metallic ions*	Control		–	100 ± 2.5
	Ca^2+^	CaCl_2_	2 mM	270 ± 6.0
	Mg^2+^	MgCl_2_	2 mM	180 ± 3.8
	Fe^2+^	FeCl_2_	2 mM	145 ± 3.4
	Cu^2+^	CuCl_2_	2 mM	99 ± 2.5
	Mn^2+^	MnCl_2_	2 mM	109 ± 2.6
	Co^2+^	CoCl_2_	2 mM	61 ± 1.2
	Ba^2+^	BaCl_2_	2 mM	75 ± 1.8
	Zn^2+^	ZnCl_2_	2 mM	40 ± 0.8
	Hg^2+^ or Cd^2+^ or Ni^2+^	HgCl_2_, CdCl_2_, or NiCl_2_	2 mM	0 ± 0.0

^a^ Values represent means of three independent replicates, and ± standard errors are reported

*SBTI* soybean trypsin inhibitor, *TPCK N*α-*p*-tosyl-l-phenylalanine chloromethyl ketone, TLCK *N*α*-p*-tosyl-l-lysine chloromethyl ketone, *DTNB* 5,5′-dithio-bis-2-nitro benzoic acid, *NEM N*-ethylmalemide, *MIA* monoiodoacetic acid, *EPNP* 1.2-epoxy-3-(*p*-nitrophenoloxy) propane, *β-ME* β-mercaptoethanol, *ld-DTT* ld-dithiothreitol

#### Effects of pH on protease activity and stability

**Fig. 3:**
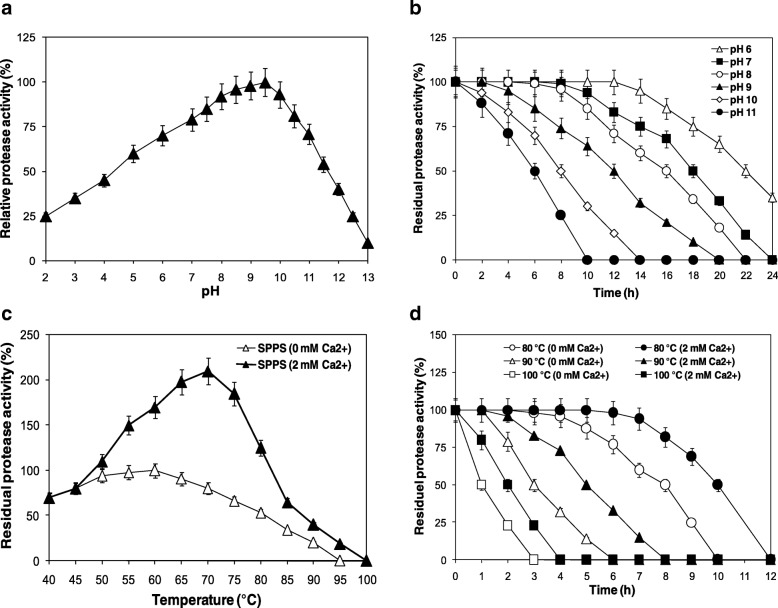
Physico-chemical proprieties of the purified protease SPPS from *Pleurotus sajor-caju* strain CTM 10057. Effects of pH on the activity (**a**) and stability (**b**) of SPPS. The activity of the enzyme at pH 9.5 was taken as 100%. Effects of the thermoactivity (**c**) and thermostability (**d**) of SPPS. The enzyme was pre-incubated for 12 h in the presence and absence of CaCl_2_ (2 mM) at temperatures ranging from 80, 90, and 100 °C. The activity of the non-heated enzyme was taken as 100%. Each point represents the mean (*n* = 3) ± standard deviation

According to the results presented in Fig. 3a, SPPS possesses an optimum activity at pH 9.5. This fungi protease keeps 60 and 71% of its original activity at pH 5.0 and 11.0, respectively. In order to study pH stability, SPPS was incubated in buffers at various pH values. The pH stability profile revealed that SPPS was highly stable in the pH range of 6.0–11.0. The half-life times of SPPS at pH 6.0, 7.0, 8.0, 9.0, 10.0, and 11.0 were 22, 20, 18, 16, 10, and 6 h, respectively (Fig. 3b). SPPS is an alkaline protease stable in alkaline solution. This propriety is solicited in protease and used as bio-additive in detergent formulations.

#### Effects of temperature on protease activity and stability

The optimum temperature for SPPS at pH 9.5 was 60 °C devoid of CaCl_2_ and 70 °C using 2 mM Ca^2+^ as shown in Fig. 3c. The half-life times of SPPS in the absence of any additive were 8, 3, and 1 h at 80, 90, and 100 °C, respectively. The addition of different concentrations of CaCl_2_ (1 to 10 mM) improved the thermostability of SPPS. The maximal thermostability was reached with 2 mM Ca^2+^. As shown in Fig. 3d, the half-life times of SPPS at 80, 90, and 100 °C increased to 10, 5, and 3 h in the presence 2 mM CaCl_2_. Compared to other proteases isolated from this genus announced in the review of Inácio et al. [1], SPPS has the highest optimum temperature and highest pH. This fungus protease is an alkaline protease acid tolerant and it is highly stable and active at high temperature and pH. SPPS is an acid resistant protease. This enzyme can have a biotechnology perspective in food applications. The high activity and stability exhibited by SPPS at high pH solutions is, in fact, a very important attribute that provides further support for its potential strong candidacy for future application in detergent formulations since laundry detergents generally operate at a pH ranging from 7.0 to11.0 [6].

#### Effect of some polyols on the thermostability of SPPS

**Fig. 4:**
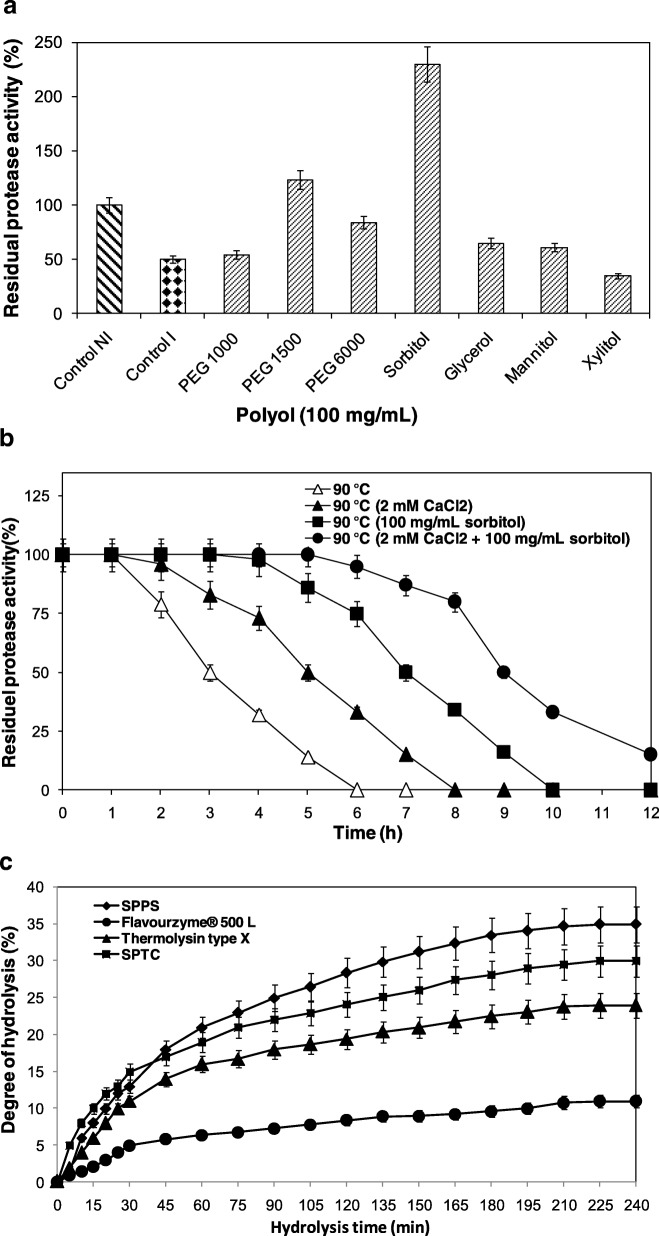
**a** Effect of polyols on protease SPPS stability. **b** The combined effect of Ca^2+^ and sorbitol 10% (v/v) on protease SPPS stability at 90 °C. **c** Hydrolysis curves of casein protein treated with various purified enzymes. The purified proteases used were: SPPS (♦), SPTC (■), Flavourzyme® 500 L (●), and Thermolysin type X (▲)

As detailed earlier, the modification of the microenvironment of enzymes via the addition of polyols in aqueous solutions generally improves its thermostability [40]. In the respect to determine the stabilizing effect, several polyols on SPPS enzyme thermal stability was investigated at 90 °C for 1 h (Fig. 3a). Data demonstrated that sorbitol was the best additive used. The combination effect of sorbitol and CaCl_2_ was also assayed at 90 °C for 12 h. The results displayed in Fig. 4b show that the half time is 9 h (in the presence of sorbitol and CaCl_2_ compared to 3 h in the absence of the two additives) (Fig. 4b). Thus, the combination effect of sorbitol and CaCl_2_ enhance the SPPS thermal stability. This improvement of SPPS thermostability in the presence of calcium and polyol is presumably attributed to the strengthening of interactions inside protein molecules and the binding of Ca^2+^ and sorbitol to the autolysis site.

### Kinetic study of the SPPS, SPTC, Flavourzyme® 500 L, and Thermolysin type X enzymes

#### Substrate specificities

The substrate specificity of proteases is often attributed to the amino-acid residues preceding the peptide bond they hydrolyze. The relative hydrolysis rates of various substrates were investigated to elucidate the amino-acid preference/substrate specificity of SPPS (Table 4). The specificity of cleavage to ester is well detailed in literature. The purified SPPS was noted to exhibit esterase and amidase activities on BTEE and ATEE, but not on TAME, BAEE, and BCEE. It also displayed a preference for aromatic and hydrophobic amino-acid residues, such as Ala, Phe, Met, Leu, and Tyr and the carboxyl side of the splitting point in the P1 position (Table 4). This indicates that the SPPS enzyme acts on hydrophobic amino–acid residues attached to the carboxyl side of the cleavage sites of the chromogenic substrate. These characteristics, were in line with the other previously depicted subtilisins from bacterial [28, 32] and fungal [26, 33, 41, 42] origins. This indicated that the SPPS protease was slightly similar to subtilisins, not only in terms of specificity for position P1, but also with regard to the effects of amino-acids residues related to the cleavage site of the enzyme.

**Table 4:** Substrate specificity profile of SPPS enzyme from *Pleurotus sajor-caju* strain CTM10057

Substrate			Concentration	Absorbance (nm) ^a^	Relative protease activity (%) ^b^
*Natural protein*	Casein		30 g/L	660	100 ± 2.5
	Gelatin		30 g/L	660	80 ± 2.0
	Keratin		30 g/L	660	37 ± 0.9
	Ovalbumin		30 g/L	660	20 ± 0.5
	BSA		30 g/L	660	10 ± 0.2
*Modified protein*	Azo-casein		25 g/L	440	100 ± 2.5
	Albumin azure		25 g/L	440	50 ± 1.8
	Keratin azure		25 g/L	595	30 ± 1.2
	Collagen type I		20 g/L	440	0 ± 0.0
	Collagen type II		20 g/L	440	0 ± 0.0
*Ester*	ATEE		10 mM	253	100 ± 2.5
	BTEE		10 mM	253	96 ± 2.4
	BAEE		10 mM	253	0 ± 0.0
	BCEE		10 mM	253	0 ± 0.0
	TAME	** ↓**	10 mM	253	0 ± 0.0
*Synthetic peptide (pNA)*		Suc-F-*p*NA	5 mM	410	49 ± 1.5
		Benz-Y-*p*NA	5 mM	410	0 ± 0.0
		M-*p*NA	5 mM	410	0 ± 0.0
		Ac-L-*p*NA	5 mM	410	0 ± 0.0
		P-*p*NA	5 mM	410	0 ± 0.0
		V-*p*NA	5 mM	410	0 ± 0.0
		Ac-A-*p*NA	5 mM	410	62 ± 1.6
		Benz-R-*p*NA	5 mM	410	0 ± 0.0
		Suc-YLV-*p*NA	5 mM	410	0 ± 0.0
		Suc-AAA-*p*NA	5 mM	410	50 ± 1.3
		Suc-AAV-*p*NA	5 mM	410	64 ± 1.7
		Suc-AAF-*p*NA	5 mM	410	62 ± 1.6
		Benz-FVR-*p*NA	5 mM	410	0 ± 0.0
		Suc-FAAF-*p*NA	5 mM	410	100 ± 2.5
		Suc-AAPF-*p*NA	5 mM	410	97 ± 2.5
		Suc-AAVA-*p*NA	5 mM	410	92 ± 2.4
		Suc-AAPM-*p*NA	5 mM	410	77 ± 1.9
		Suc-AAPL-*p*NA	5 mM	410	65 ± 1.7
		Suc-LLVY-*p*NA	5 mM	410	57 ± 1.6
		Ac-YVAD-*p*NA	5 mM	410	0 ± 0.0

^a^ Values represent means of three independent replicates, and ± standard errors are reported

^b^ The activity of each substrate was determined by measuring absorbance at specified wave lengths according to the relative method reported elsewhere [33, 34]

BTEE, *N*-benzol-l-tyrosine ethyl ester; ATEE, *N*-acetyl-l-tyrosine ethyl ester monohydrate; BAEE, *N*-benzol-l-arginine ethyl ester; BCEE, *S*-benzyl-l-cysteine ethyl ester hydrochloride; TAME, *N*_α_-*p*-tosyl- l-arginine methyl ester hydrochloride; *N*-succinyl-F-*p*-nitroanilide; *N*-benzoyl-Y-*p*-nitroanilide; M-*p*-nitroanilide; *N*-acetyl-L-*p*-nitroanilide; P-*p*-nitroanilide trifluoroacetate salt; V-*p*-nitroanilide hydrochloride; *N*-acetyl-A-*p*-nitroanilide; *N*-benzoyl-R-*p*-nitroanilide; *N*-succinyl-YLV-*p*-nitroanilide; *N*-succinyl-AAA-*p*-nitroanilide; *N*-succinyl-AAF-*p*-nitroanilide; *N*-succinyl-AAV-*p*-nitroanilide; *N*-benzoyl-FVR-*p*-nitroanilide; *N*-succinyl-FAAF-*p*-nitroanilide; *N*-succinyl-AAPF-*p*-nitroanilide; *N*-succinyl-AAVA-*p*-nitroanilide; *N*-succinyl-AAPM-*p*-nitroanilide; *N*-succinyl-AAPL-*p*-nitroanilide; *N*-succinyl-LLVY-*p*-nitroanilide; and *N*-acetyl-YVAD-*p*-nitroanilide

#### Determination of kinetic parameters

The kinetic parameters determined from Lineweaver–Burk plots are shown in Table 5 for SPPS, SPTC, Flavourzyme® 500 L, and Thermolysin type X purified enzymes using casein as substrate. The four used enzymes exhibited the classical kinetics of Michaelis–Menten. The returns indicate that the *K*_*m*_ and *V*_max_ parameters for the SPPS enzyme, measured with casein, were 0.295 mg/mL and 79,000 μmol/mg.min^− 1^. The deduced catalytic efficiency (*k*_cat_/*K*_m_) of the SPPS enzyme is 1.45, 3.33, and 8.48 times compared to SPTC; Flavourzyme® 500 L, and Thermolysin type X, respectively.

**Table 5:** Kinetic parameters of the purified proteases: SPPS, SPTC, Flavourzyme® 500 L, and Thermolysin type X for hydrolysis of natural protein (casein)

Enzyme	Origin	*K*_*m*_ (mg/mL) ^a^	*V*_*max*_ (μmol/mg.min^−1^) ^a^	*k*_*cat*_ (min^−1^)	*k*_*cat*_/*K*_*m*_ (mL.mg^−1^.min^−1^)
SPPS	*Pleurotus sajor-caju* CTM 10057	0.275 ± 0.01	79,000 ± 695	52,667	191,516
SPTC	*Trametes cingulata* CTM10101	0.475 ± 0.02	94,075 ± 856	62,717	132,036
Flavourzyme® 500 L	*Aspergillus oryzea*	0.546 ± 0.04	47,125 ± 566	31,417	57,540
Thermolysin type X	*Geobacillus stearothermophilus*	0.613 ± 0.05	20,764 ± 323	13,843	22,582

^a^ Values represent means of three independent replicates, and ± standard errors are reported

The protease from *Pleurotus sajor-caju* strain CTM10057 displayed a *K*_*m*_ value (0.275 mg/mL).

### Performance evaluation of the purified SPPS, SPTC, Flavourzyme® 500 L, and Thermolysin type X enzymes

#### Determination of hydrolysis degree

Enzymatically hydrolyzed proteins possess functional properties, such as low viscosity, increased whipping ability, and high solubility, which make them favorable for food industry uses [43]. The hydrolysis degree of SPPS is achieved in curves of casein protein, after 240 min of incubation are shown in Fig. 4c. The purified enzymes were used at the same levels of activity for the production of protein hydrolysates from casein and for the subsequent comparisons of hydrolytic efficiencies. As shown in Fig. 4c, the purified SPPS was the most efficient, with 35% protease used during hydrolysis, followed by SPTC (30%) and Thermolysin type X (24%) with Flavourzyme® 500 L being the least efficient (11%) one.

These findings indicate that SPPS enzyme can be usefully employed for the preparation of protein hydrolysates.

#### Effects of organic-solvents on protease activity and stability of SPPS and Thermolysin type X

**Fig. 5:**
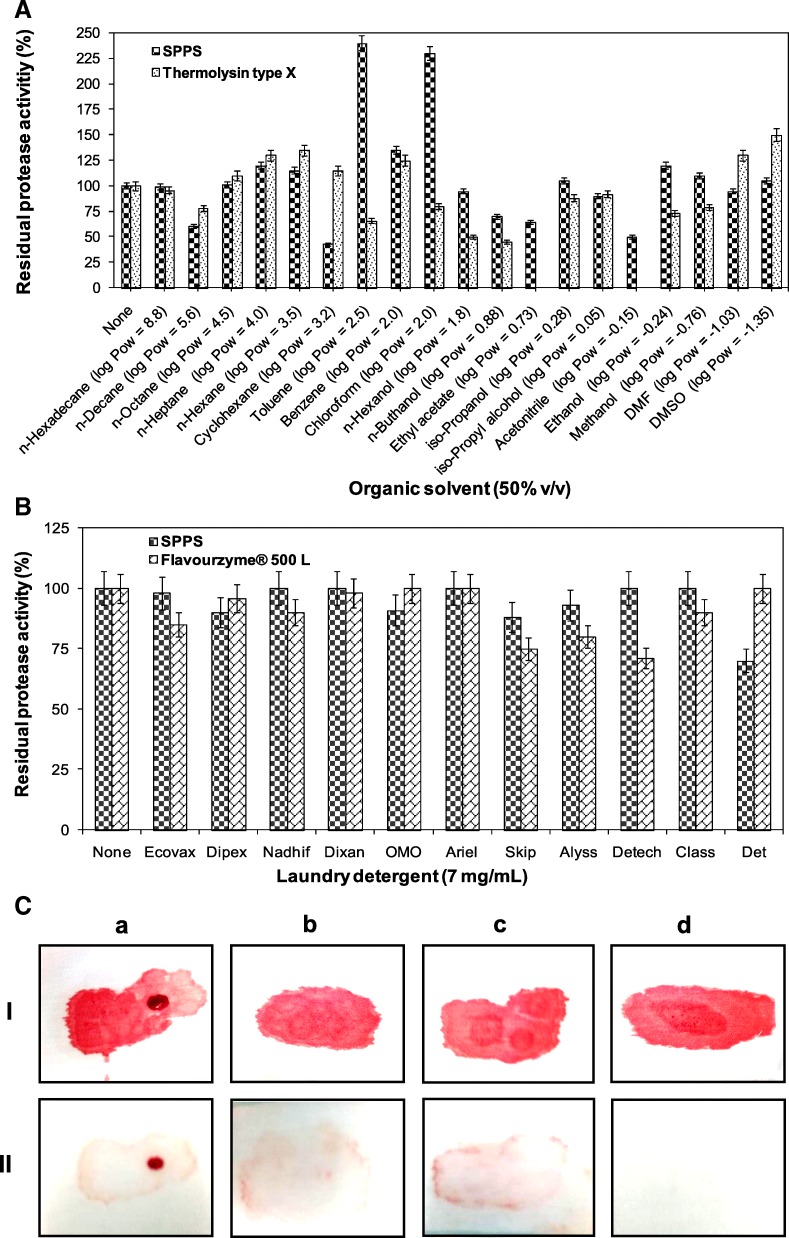
**A** Effect of different organic solvent on SPPS and Thermolysin type X enzyme stabilities; **B** Stability of SPPS and Flavourzyme® 500 L purified proteases in the presence of commercial liquid [Nadhif from Henkel-Alki (Tunisia), Ariel from Procter & Gamble (Switzerland), Skip from Unilever (France), Class from EJM (Tunisia), and Dipex from Klin Productions (Tunisia)] and solid [Dixan from Henkel (France), Ecovax from Klin Productions (Tunisia), OMO from Unilever (France), Alyss from EJM (Tunisia), Detech from SOTUP (Tunisia), and Det from Sodet (Tunisia)] laundry detergents at 7 mg/mL. Enzyme activity of the control sample, which contained no additive and incubated under similar conditions, was taken as 100%. Vertical bars indicate standard error of the mean (*n* = 3). **C** Washing performance analysis test of SPPS enzyme in the presence of the commercial detergent Dixan. Cloth stained with blood washed with: (**a**) tap water, (**b**) Dixan detergent (7 mg/mL), (**c**) Dixan added with Flavourzyme® 500 L (commercial enzyme, 500 U/mL), and (**d**) Dixan added with SPPS (500 U/mL). I: untreated cloths (control) and II: treated cloths

The effects of various organic solvents (at 50% v/v, with different log P_ow_) on the stability of the purified SPPS and Thermolysin type X enzymes were determined by measuring residual activity under the optimum condition of each used enzyme after 72 h of incubation at 37 °C (Fig. 5a). When it is compared to Thermolysin type X, SPPS is stable in both hydrophobe and hydrophile organic solvents. It was also noted to be more stable and tolerant in the presence of the acetonitrile. The SPPS protease has then an elevated tolerance to organic solvents, whatever their hydrophobicity is. It can be used yet in homogeneous and heterogeneous catalysis. It was well informative that enzyme deactivation can be assigned to the change of medium hydrophobicity [44]. Logically, polar solvent can penetrate into protein and changing structural for the interaction between the active site and substrate than non polar solvents. Thus, SPPS can be active in aqueous and non aqueous solutions. Accordingly, SPPS can be a potential candidacy for future application as a biocatalyst for the synthesis of peptide reactions in low water activity systems.

#### Stability and compatibility of SPPS and Flavourzyme® 500 L with laundry detergents

Recent researches have aimed at finding a substitute for the costly toxic chemicals by bio-products. Correspondingly, the use of proteases in the detergent field provides environmental and energetic advantages. In terms of energy, proteases use consists in washing at lower temperatures (10–20 °C). Moreover, the environmental benefit of such proteases incorporates less chemical surfactants use in order to preserve the environment. However, the desired detergent protease must be very active and stable in alkaline solution with respect to detergents [2]. This experience explores the application of SPPS protease in detergent industry. The data presented in Fig. 5b show that, compared to Flavourzyme® 500 L, SPPS was extremely stable and compatible with the commercial liquid detergents used, retaining 100% of its initial activity with Dixan, Nadhif, Detch, and Class detergents at a concentration of 7 mg/mL vs 100% of the initial activity of Flavourzyme® 500 L with OMO, Ariel, and Det.

These results can approve the usefulness of SPPS as a cleaning bio-additive in detergent formulations used.

#### Cleaning potential of detergent additive SPPS to remove the blood strain from cotton fabrics

Alkaline serine proteases are usually sought in detergence industry [45]. They highly increase the cleaning efficacy of laundry detergent. Accordingly, a comparative test using Flavourzyme® 500 L in Dixan was carried out (Fig. 5c). In fact, the blood strain was completely removed. It is noted that SPPS isolated from *Pleurotus sajor-caju* strain CTM10057 is more efficient than Flavourzyme® 500 L in Dixan. Thus, SPPS can be considered as a remarkable bio-additive in Dixan.

## Discussion

This study is concerned with the production, purification and characterization of a new serine fungi protease. This protease is produced by *Pleurotus sajor-caju* strain CTM10057. The purification procedure strated by heat treatment (80 °C for 20 min) followed by ammonium sulfate precipitation (35–55%)-dialysis, then UNO Q-6 FPLC ion-exchange chromatography and finally HPLC-ZORBAX PSM 300 HPSEC gel filtration chromatography. SPPS contained about 15% of the total activity of the crude and had a specific activity of 79,000 U/mg (Table 1) using casein as substrate. This specific activity is higher than *Aspergillus oryzae* strain CH 93 (15,86 U/mg) [14] and *Aspergillus clavatus* strain ES1 (37,600 U/mg) [25]. According to native and SDS page study, SPPS was a homogeneous enzyme with high purity as it exhibits a unique elution symmetrical peak at retention time (R_t_) = 11.6 min corresponding to a single protein of nearly 65 kDa by HPLC gel filtration chromatography (Fig. 2b). This molecular weight was higher compared to those previously mentioned with respect to other fungal proteases like *Pleurotus sajor-caju* (48 kDa) [39]; *Pleurotus citrinopileatus* (28 kDa) [46]; *Aspergillus clavatus* ES1 (30 kDa) [25], and *Aspergillus oryzae* CH93 (47.5 kDa) [14]. Moreover, the electrophoretic analyses and size exclusion chromatography showed that SPPS is a monomer comparable to the other previously reported fungi proteases [25, 26, 33, 39]. These results strongly suggested that SPPS enzyme from strain CTM10057 was a novel protease. The highest homology was noted with the protease isolated from *Pleurotus* genus (90%). SPPS belongs to the serine protease family according to the inhibitory test in bibliography, 22% of basidiomycete’s protease belongs to serine protease [19]. Besides, this protease’s class is widespread in eukaryotes microorganisms [13]. This enzyme is totally activated by Ca^2+^, Mg^2+^, and Fe^2+^, however, strongly inhibited by Cd^2+^, Ni^2+^, and Hg^2+^ (2 mM). This is similar to other serine proteases such as ES1 [25], SAPB [32], serine protease isolated from *Pleurotus sajor-caju* increased by Ca^2+^ and Mg^2+^ [39], protease isolated from *Aspergillus oryzae* strain CH93 and protease isolated from *Aspergillus tamari* strain URM 4634 [47]. Serine proteases are used to incorporate two calcium binding sites [34, 48] and their addition increase thermostability. It plays an important role in maintaining their 3D structure. In fact, Ca^2+^ was previously reported to improve serine protease activity and stability [32]. These ions can preserve the active confirmation at higher temperature [49]. This kind of protease (serine protease) is well known in the field of detergency. Insensitivity to chelators would be a valuable property for potential application in detergent formulations since these agents are added to soften water and remove stain [6]. The SPPS activity was totally inhibited by mercury ions, which could be ascribed to the exert toxicity of these ions, especially towards thiol serine protease [50, 51].

SPPS is an alkaline protease stable in alkaline solution. This propriety is also searched in protease and used as bio-additive in the detergent preparation. Proteolysis enzymes with appropriate specificity and stability to temperature and pH are necessary for the physiology of ligninolytic fungi [52]. The thermo-stability of SPPS in very high temperature is a substantial feature preferred in proteases used as bio-additive in laundry detergent [9]. Extremely high temperature can destabilize the non-covalent interactions of protein [8, 53]. It has further been suggested that the protective role of polyols is ascribed to their capability to form hydrogen bonds that support and stabilize the native conformation of the enzyme to make it more resistant to heat treatment. The impact of polyols on protease activity is well studied. Other researchers have also shown the improvement of thermostability of proteases in the presence of polyols [28, 54]. The protective impact of polyols is dispersed based on these latter’s hydroxyl groups belonging. The addition of some polyols is pivotal in order to maintain hydrophobic interactions within protein molecules. It has further been suggested that the protective role of polyols is assigned to their capability to form hydrogen bonds that support and stabilize the native conformation of the enzyme to make it more resistant to heat treatment.

The stability of microbial proteases by polyols has been broadly depicted by several works, such as SPACG from *Caldicoprobacter guelmensis* strain D2C22^T^ [54], and SAPHM from *Bacillus licheniformis* strain K7A [33]. The highest activity on protein was noted with casein, followed by gelatin, and albumin azure. Other serine proteases such as BAP from *Beauveria* sp. strain MTCC 5184 possess the same substrate specificity. It was more active against casein compared to hemoglobin and bovine serum albumin [51]. This specificity of cleavage to natural protein has already been elaborated in other proteases from fungi. The substrate specificity profile of SPPS indicates that it is subtilisin-like. However, the enzyme activity of protease produced by *Aspergillus flavus* strain AP2 was the highest with gelatin [55]. The protease from *Pleurotus sajor-caju* strain CTM10057 has a less *K*_*m*_ value (0.275 mg/mL) as proteases isolated from *Pleurotus citrinopileatus* (3.44 mg/mL) [46], *Amanita virgineoides* (3.74 mg/mL) [56], and *Helvella lacunose* (3.81 mg/mL) [57], i.e. SPPS protease evinced a higher affinity towards casein than the above-mentioned proteases. Nevertheless, SPPS has a *K*_*m*_ value (0.275 mg/mL) comparable with the protease isolated from *Mucor bacilliformis* (*K*_*m*_ = 0.185 mg/mL) [58] and *Cordyceps sobolifera* (0.41 mg/mL) [59], which means similar affinities towards casein possessed three fungal proteases. Hopefully, future works can find out the position of substrate binding site and modify it to enhance the substrate affinity. Thereby, the presence of some organic solvents altering the structure-function of many proteins [60]. As well detailed, the proteases produced by *Pseudomonas aeruginosa* PseA and PST-01 have a high stability and tolerance toward hydrophobic and hydrophilic solvents, respectively [61]. However, Jaouadi et al. reported that AMPP protease produced by *Pseudomonas aeruginosa* CTM50182 displayed high stability in the presence of various organic solvents than commercial enzymes [62]. On the other hand, *n*-butanol showed strong inhibition in protease activity from *Aspergillus niger* WA 2017 [63]. Thus, the intrinsic organic solvent-tolerance and stability of some enzymes depends on the nature of the used organic solvents, which makes them highly attractive to industry [64, 65].

Owing to its stability towards detergency compositions, SPPS can be used in detergent formulations as a cleaning bio-additive. A similar work has shown the stability and compatibility of the protease from *Aspergillus terreus* gr. in the presence of some comment ingredient bleach-based detergent formulation [16], showing that it is appropriate candidate for cleaning power of laundry detergent compositions [66] . The alkaline protease isolated from *Pleurotus sajor-caju* CTM10057 was completely stable in the presence of various detergent additives than Flavourzyme® 500 L. Similar studies have revealed that alkaline proteases produced by *Bacillus licheniformis* [33], *Aspergillus terreus* [16], and *Aspergillus niger* [67] demonstrated good stability in commercial laundry detergents. In alkaline condition, the stability of the protease SPPS indicates a significant enzyme compatibility with laundry detergent and, therefore, making it a potential candidate for use in detergent formulations as cleaning additive that facilitates proteinaceous stains release.

## Conclusions

Accordingly, a novel fungal protease with a great industrial interest, from *Pleurotus sajor-caju* CTM 10057 was purified to homogeneity and biochemically characterized. Therefore, this mushroom constitutes a new source of proteolytic activity for future applications. Compared to SPTC and commercial proteases Flavourzyme® 500 L and Thermolysin type X, SPPS showed high hydrolysis degree and catalytic efficiency. Besides, compared to Thermolysin type X and Flavourzyme® 500 L, SPPS displayed significant tolerance in the presence of some organic solvents, as well as an excellent stability and compatibility with a wide range of commercialized laundry detergents, respectively. Thus, SPPS offers new and promising opportunities for industrial and biotechnological perspective bioprocess, mainly in peptides synthesis and detergent formulation. Further and supplementary works are needed to isolate the *spPS* encoding gene, to hyperproduce the recombinant enzyme (rSPPS) in heterologous-expression system and to explore its structure**-**function relationships using site**-**directed mutagenesis approach and 3D structure modeling.

## Data Availability

The datasets generated and/or analyzed during the current study are available on the GenBank repository, https://www.ncbi.nlm.nih.gov/genbank/. The GenBank accession number for the nucleotide sequence of the 18S rDNA gene referred to in the text is MH806376. Other datasets generated during and/or analyzed during the current study available from the corresponding author on reasonable request.

## Abbreviations

ATEE: *N*-acetyl-l-tyrosine ethyl ester monohydrate
BAEE: *N*-benzyol-l-arginine ethyl ester
BCEE: *S*-benzyl-l-cysteine ethyl ester hydrochloride
BSA: Bovine serum albumin
BTEE: *N*-benzol-l-tyrosine ethyl ester
DFP: Diiodopropyl fluorophosphate
DMC: *N*,*N*-dimethylated casein
DTNB: 5,5′-dithio-bis-2-nitro benzoic acid
EPNP: 1.2-epoxy-3-(*p*-nitrophenoloxy) propane
FPLC: Fast protein liquid chromatography
HPLC: High performance liquid chromatography
iodoacetamide: β-ME, β-mercaptoethanol
ld-DTT: ld-dithiothreitol
MIA: Monoiodoacetic acid
NEM: *N*-ethylmalemide
PAGE: Polyacrylamide gel electrophoresis
PCR: Polymerase chain reaction
PDB: Potato dextrose broth
PEG: Propylene glycol
PMSF: Phenylmethyl sulfonyfluoride
rDNA: ribosomal DNA
SBTI: Soybean trypsin inhibitor
SDS: Soduim dodecyl sulphate
SPPS: Serine alkaline protease from *Pleurotus sajor-caju* strain CTM10057
TAME: *N*_α_-*p*-tosyl-l-arginine methyl ester hydrochloride
TLCK: *N*α*-p*-tosyl-l-lysine chloromethyl ketone
TPCK: *N*α-*p*-tosyl-l-phenylalanine chloromethyl ketone
